# RNA-Seq Provides New Insights into the Gene Expression Changes in *Azoarcus olearius* BH72 under Nitrogen-Deficient and Replete Conditions beyond the Nitrogen Fixation Process

**DOI:** 10.3390/microorganisms9091888

**Published:** 2021-09-06

**Authors:** Shanmugam Solaiyappan Mani, Barbara Reinhold-Hurek

**Affiliations:** Department of Microbe-Plant Interactions, Faculty of Biology and Chemistry, University of Bremen, P.O. Box 330440, 28334 Bremen, Germany; s_ado8ut@uni-bremen.de

**Keywords:** biological nitrogen fixation, RNA-Seq, endophyte

## Abstract

*Azoarcus olearius* BH72 is an endophyte capable of biological nitrogen fixation (BNF) and of supplying nitrogen to its host plant. Our previous microarray approach provided insights into the transcriptome of strain BH72 under N_2_-fixation in comparison to ammonium-grown conditions, which already indicated the induction of genes not related to the BNF process. Due to the known limitations of the technique, we might have missed additional differentially expressed genes (DEGs). Thus, we used directional RNA-Seq to better comprehend the transcriptional landscape under these growth conditions. RNA-Seq detected almost 24% of the annotated genes to be regulated, twice the amount identified by microarray. In addition to confirming entire regulated operons containing known DEGs, the new approach detected the induction of genes involved in carbon metabolism and flagellar and twitching motility. This may support N_2_-fixation by increasing energy production and by finding suitable microaerobic niches. On the other hand, energy expenditures were reduced by suppressing translation and vitamin biosynthesis. Nonetheless, strain BH72 does not appear to be content with N_2_-fixation but is primed for alternative economic N-sources, such as nitrate, urea or amino acids; a strong gene induction of machineries for their uptake and assimilation was detected. RNA-Seq has thus provided a better understanding of a lifestyle under limiting nitrogen sources by elucidating hitherto unknown regulated processes.

## 1. Introduction

Nitrogen, a component of biomolecules such as proteins and nucleic acids, is important for the growth of plants [1]. In spite of its abundance in the atmosphere, plants cannot directly assimilate N_2_ and depend on the combined nitrogen sources from soil. Only certain prokaryotes have the capability to convert atmospheric N_2_ into combined forms for plant assimilation. This process is termed biological nitrogen fixation (BNF), and the prokaryotes that perform BNF are referred to as diazotrophs. Harnessing the power of BNF grants us a better alternative for chemical fertilizers, thereby taking us a step closer to sustainable agriculture [2]. Our model organism, *Azoarcus olearius* BH72, is a diazotroph belonging to the class *Betaproteobacteria* [3]. It is an endophyte that was isolated from the surface-sterilized roots of Kallar grass (*Leptochloa fusca* L. Kunth) from the Punjab region of Pakistan [4]. Earlier, it was confirmed that strain BH72 supplies fixed nitrogen to its host plant, Kallar grass [5]. This strain has been shown to colonize rice roots and endophytically express nitrogenase genes under gnotobiotic conditions within rice roots [6,7]. Another strain of this species, DQS-4, isolated from oil-contaminated soil in Taiwan can fix atmospheric nitrogen as well [8]. This strain is also able to colonize rice roots endophytically and has exhibited plant growth-promoting activity [9]. Hence, studying gene regulation in strain BH72 under BNF will strengthen our understanding of specific requirements and activities of *A. olearius* while fixing nitrogen.

As an initial step in this quest, we previously used microarrays to compare the transcriptome of strain BH72 under N_2_-fixing and ammonium-grown conditions [10]. For this analysis, we used transcriptome microarrays spotted with 70 mer oligonucleotide probes as quadruplicates for the 3989 predicted protein-coding genes from *A. olearius* BH72 [10]. As expected, this study revealed several BNF-related genes to be differentially expressed. This included the genes encoding for the transcriptional regulator of BNF (NifA), components of nitrogenase, its maturation factors, and the energy transfer system (Rnf1 complex). Simultaneously, it elucidated the regulation of processes that did not have a direct link to BNF such as type 2 and one of the two type 6 protein secretion systems (T2SS and T6SS-1). The T6SS is present in a wide range of endophytes [11], and especially in symbiotic rhizobia, the T6SS-1 (Imp system) affected the host range of nodulation [12]. Therefore, in strain BH72, they may play an important role in plant colonization as well. Presumably, the bacterium already induces their expression under BNF as a preparatory step for its lifestyle. Remarkably, the microarray study was the first to report a BNF-dependent induction of T6SS expression [10,13]. Nevertheless, microarrays failed to detect genes that were previously shown to be differentially expressed under similar growth conditions such as *nifH*, *fdxN*, *draG*, *amtB*, and *amtE* [7,14,15,16,17]. This phenomenon could be attributed to the known limitations of the microarray approach, such as lower detection range, cross-hybridization, non-specific hybridization, and spot inhomogeneity. These limitations might have prevented microarrays from identifying other vital components and regulations during BNF. This additional knowledge will help in answering several open questions. One of the persisting questions pertains to how *Azoarcus* provides the necessary energy for BNF. A recent study has revealed that in *E. coli* that has been artificially engineered to perform BNF, the metabolic flux shifted from the tricarboxylic acid (TCA) cycle to the pentose phosphate pathway (PPP) and to ethanol fermentation to support BNF [18]. In strain BH72, microarrays detected the genes involved in the Embden–Meyerhof–Parnas pathway to be repressed but did not detect differential regulation in any of the alternate metabolic pathways to sustain the energy supply.

RNA-Seq overcomes many of the above limitations of microarrays. RNA-Seq has a higher sensitivity, specificity, and increased dynamic range of detection. Additionally, studies have already shown that RNA-Seq approaches reveal information and regulatory features in addition to the data from microarray analysis [19,20,21,22,23,24]. Consequently, RNA-Seq has superseded microarrays for transcriptome profiling. Nevertheless, the application of RNA-Seq to understand BNF is scarce. In *Azotobacter vinelandii,* almost 30% of the annotated genes were found to be differentially expressed during N_2_-fixing growth in comparison to non-fixing growth [25]. The quantity was even higher for *Paenibacillus* sp. WLY78; roughly 60% of the genes were detected to be regulated [26]. These are remarkably higher compared to our previous microarray analysis (13%). This highlights the depth of knowledge that could be obtained by implementing RNA-Seq to identify genes regulated under BNF in strain BH72. Hence, in this study, we ventured to analyze the transcriptome of strain BH72 during diazotrophic growth in comparison to ammonium-grown conditions using directional RNA-Seq.

## 2. Materials and Methods

### 2.1. Growth and Harvesting of Bacterial Cultures

*Azoarcus olearius* strain BH72 was initially grown in minimal medium (synthetic malate, SM) [27] without biotin. The cells were washed thrice with nitrogen-free SM-medium and were used as an inoculant. For the N_2_-fixing conditions, strain BH72 was cultivated microaerobically (0.3% O_2_) in a bioreactor (Minifors, Infors HT, Einsbach, Germany) at 37 °C with constant stirring at 600 rpm in 1.6 L of N-free SM medium in the presence of N_2_. As a control, the strain was also grown in ammonium-based conditions by supplementing the SM medium with 10 mM NH_4_Cl. Under both conditions, three independent cultures were set up. An amount of 30 mL of the cultures was harvested at an optical density (OD_578_) of 0.5–0.7 by centrifuging at 5500× *g* and 25 °C for 10 min. The supernatant was removed, and the pellets were frozen in liquid nitrogen and were stored at −80 °C.

### 2.2. Extraction of RNA from Bacterial Cells

RNA extraction was performed by means of the hot phenol method, as described previously [10]. In brief, frozen cell pellets were re-suspended in 8 mL of the hot phenol solution. The hot phenol solution was prepared by mixing equal quantities of phenol–chloroform–isoamyl alcohol (25:24:1 (*v/v/v*)) (pH 4.0) (AppliChem GmbH, Darmstadt, Germany; CAS registry numbers 108-95-2, 67-66-3 and 123-51-3, respectively) and NAES (50 mM Sodium acetate, 10 mM EDTA, and 1% (*w/v*) SDS at pH 5.1) and was heated to 65 °C. The cell suspension was incubated at 65 °C for 5 min, further incubated on ice for 10 min, and then centrifuged at 13,000× *g* and 12 °C for 15 min. The aqueous phase was extracted again with an equal volume of phenol–chloroform–isoamyl alcohol (pH 4.0), and this step was repeated thrice. The resulting aqueous phase was mixed with an equal volume of a chloroform–isoamyl alcohol mixture (24:1 (*v/v*)) (Carl Roth GmbH, Karlsruhe, Germany, CAS registry numbers 67-66-3 and 123-51-3, respectively) and was extracted after centrifugation at 6300× *g* and 12 °C for 5 min. After extraction, the nucleic acids were precipitated with an equal volume of isopropanol (VWR International GmbH, Darmstadt, Germany; CAS registry number 67-63-0), were incubated on ice for 60 min, and were centrifuged at 13,000× *g* and 4 °C for 10 min. The pellet was washed with the same volume of 70% ethanol (VWR International GmbH, Darmstadt, Germany; CAS registry number 64-17-5) and was centrifugated at 4 °C for 5 min. After drying, the pellet was re-suspended in 30 µL of RNase free water. The sample was immediately frozen in liquid nitrogen and was stored at −80 °C for further processing.

From the extracted nucleic acids, genomic DNA was eliminated using the RNeasy Plus Mini Kit (Qiagen, Hilden, Germany, Catalog no. 74134) as per the protocol prescribed by the manufacturer for the purification of total RNA containing small RNAs. In the procedure, 10 µg of the extracted nucleic acid was used as the sample instead of cells.

### 2.3. Preparation and Sequencing of Directional RNA-Seq Library

The RNA samples were further processed and sequenced at Vertis Biotechnologie AG, Freising, Germany. The ribosomal RNA was depleted using the Ribo-Zero rRNA Removal Kit for bacteria (Illumina, Berlin, Germany, Catalog no. MRZB12424). The samples were fragmented by four pulses of ultrasound that were 30 s each at 4 °C. To aid in directional sequencing, a 3′ Illumina TruSeq sequencing adapter with barcodes was ligated to the 3′ end of the fragmented RNA molecules. The first-strand cDNA was synthesized using M-MLV reverse transcriptase and a primer binding to the adapter sequence. Synthesized cDNA was purified, and to its 3′ end, the 5′ Illumina TruSeq sequencing adapter, which also contained barcodes, was ligated. The cDNA was amplified through 13 cycles of PCR using a high-fidelity DNA polymerase to obtain about 10–20 ng/µL of DNA. The amplified cDNA was purified using an Agencourt AMPure XP kit (Beckman Coulter Genomics, Krefeld, Germany). The six samples were pooled in approximately equimolar amounts and were size-fractionated using a preparative agarose gel to obtain a size range between 200 and 400 bp. The sample pool was sequenced on an Illumina NextSeq 500 system using a 75 bp read length. The sequence of the TruSeq adapters and the barcodes used in this procedure are provided in Tables S1 and S2, respectively. The reads obtained from the RNA-Seq have been deposited in the NCBI’s Gene Expression Omnibus [28] and are accessible through the GEO Series accession number GSE176473 (https://www.ncbi.nlm.nih.gov/geo/query/acc.cgi?acc=GSE176473).

### 2.4. Mapping of Reads to the Genome Sequence and Analysis of Differential Expression

The Illumina sequencing reads were trimmed using the fastptool [29] based on the adapter sequence (AGATCGGAAGAGCACACGTCTGAACTCCAGTCA) and the read quality. Reads with at least one “N” base or more than 10 bases with a Phred score less than 10 were removed. From the 5′ and 3′ end of the reads, bases up to a 3-nucleotide window that had a mean quality score less than 10 were also dropped. Processed reads were aligned to the genome sequence of *Azoarcus olearius* strain BH72 using the READemption tool [30] with its default parameters. Only reads that mapped uniquely were considered for quantification. Raw coverage values for the annotated CDSs were rounded and were used for differential expression analysis using the DEseq2 tool [31]. In the tool, the results were optimized for an FDR cutoff value of 0.05 by means of the independent filtering of the genes based on mean normalized counts.

### 2.5. Verification of RNA-Seq Data by Quantitative RT-PCR

The differential expression of selected targets identified by RNA-Seq was verified through quantitative RT-PCR of RNA samples. For each target, the first-strand cDNA was synthesized by Superscript III reverse transcriptase (ThermoFischer Scientific, Darmstadt, Germany, Catalog no. 18080093). Specifically, 2.5 µM of gene-specific reverse primer and 20 ng of total RNA were used in a 10 µL reaction volume and were incubated at 55 °C. Other components and steps were followed as per the prescribed protocol. In each reaction, 10 U of Recombinant RNasin Ribonuclease Inhibitor (Promega, Walldorf, Germany, Catalog no. N2515) was added instead of RNaseOUT. For quantification, 1 µL from the reverse transcription reaction was further amplified using SsoAdvanced Universal SYBR Green Supermix (Bio-Rad Laboratories, Feldkirchen, Germany, Catalog no. 1725271) with 0.3 µM of both forward and reverse primers in a 20 µL reaction volume. The reaction was initiated with an initial denaturation (98 °C for 2 min) followed by 40 cycles of two-step amplification (10 s each at 98 °C and respective annealing temperatures) in a CFX96 Real-time PCR Detection System (Bio-Rad Laboratories, Feldkirchen, Germany). The melting curve of the product was analyzed by heating the samples from 65 °C to 95 °C in 0.5 °C increments with a 5 s incubation period at each step. The PCR efficiency was verified using the LinRegPCR tool [32]. Relative change in the expression of the targets was calculated through the ‘delta-delta Ct’ method using *gapA* gene (*azo2837*) as the internal reference. A list of gene-specific primers and annealing temperatures for each pair are provided in Table S1.

## 3. Results and Discussion

### 3.1. New Information on the Transcriptome under N_2_-Fixing Conditions Provided by RNA-Seq Compared to Previous Microarray-Based Transcriptome Analyses

In this study, we performed a directional RNA-Seq analysis to compare the transcriptome of diazotrophic *Azoarcus olearius* BH72 grown microaerobically under N_2_-fixing and non-fixing conditions (with ammonium chloride as nitrogen source). Cells and RNA were obtained from three independent experiments for each condition. The RNA-Seq approach yielded more than 10 million reads for each processed sample. Out of these, 83–88% of the reads aligned to the genome of *Azoarcus* (Table S3). For this study, we only considered the reads that aligned uniquely along the sense strand of the annotated genes. Under N_2_-fixing and ammonium-grown conditions, 3221 and 3094 open reading frames (ORFs), respectively, were detected to be transcribed with a minimum reads per Kilobase per Million mapped reads (RPKM) value of 10 in all three biological replicates. Together, this constituted 3300 unique ORFs (82% of the 3989 annotated ORFs). As expected, the Euclidean distance between the samples, using the count data after variance-stabilized transformation, indicated a closer relationship between the biological replicates grown in the same conditions compared to the distance between the replicates from different growth conditions (Figure S1).

Using the DESeq2 tool [31], differential expression analysis of the data revealed 966 genes (24% of the annotated genes) to be significantly regulated with the criteria of a fold-change more than 1.8 and a Benjamini–Hochberg adjusted *p*-value (padj) less than 0.05 (Table S4). Out of these, 533 and 433 genes were up-and down-regulated under N_2_-fixing conditions, respectively. Only a couple of previous studies have utilized similar RNA-Seq platforms to explore the transcriptome under diazotrophic growth. In the Gram-negative bacterium *A. vinelandii*, almost 30% of the annotated genes were identified to be differentially regulated (fold-change > 2 and adjusted *p*-value < 0.01) under N_2_-fixing conditions [25]. In *Paenibacillus* sp. WLY78, an astounding 60% of the annotated genes were reported to be differentially regulated (fold-change > 2 and *p*-value < 0.05) under N_2_-fixing conditions [26]. In contrast to our analysis, this study used aerobically (21% O_2_) grown culture as the control. Hence, a considerable portion of the genes that were detected to be differentially expressed might be functionally important for growth at high versus low oxygen concentrations. In a microarray-based transcriptome study in *Azoarcus*, we showed that at least 8.7% of the annotated ORFs were differentially expressed during microaerobic growth when compared to aerobic conditions [33].

**Figure 1:**
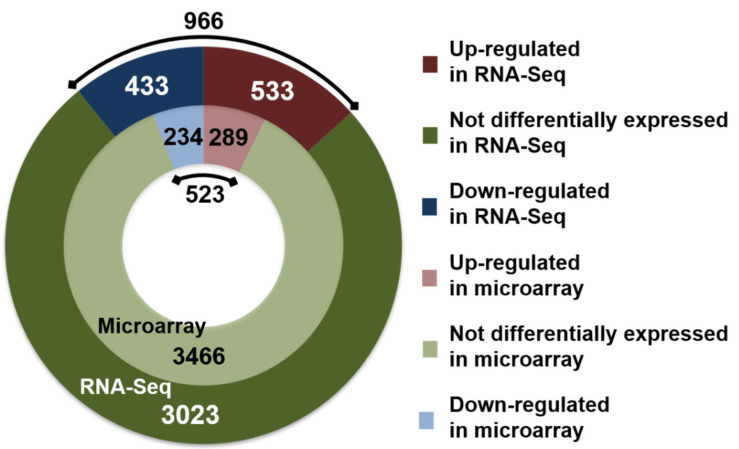
Number of differentially expressed genes (DEGs) detected in *A. olearius* BH72 under N_2_-fixing versus ammonium-based growth conditions. Quantitative comparison of DEGs detected by RNA-Seq (this study) in comparison to previous microarray analysis [10]. Genes with a fold change > 1.8 and padj < 0.05 were regarded as DEGs. Up- or down-regulation refer to the enhanced or suppressed expression, respectively, detected under N_2_-fixing conditions in comparison to growth in ammonium. The black arc (with numbers) represents the total number of DEGs.

We had previously compared the transcriptome of N_2_-fixing and ammonium-grown *A. olearius* using the microarray approach under otherwise comparable experimental conditions [10]. This study identified 523 genes to be differentially regulated. Roughly, twice this quantity has been identified by the current RNA-Seq approach (Figure 1). As shown in Figure S2, 751 genes were uniquely identified using RNA-Seq alone. These additional data shed a brighter light on the currently unknown differentially expressed genes (DEGs) during the diazotrophic growth of *A. olearius*. DEGs uniquely identified by RNA-Seq included genes that encoded for proteins involved in vital pathways such as those for nitrogen assimilation, energy production, motility, and vitamin biosynthesis.

Along with these DEGs, RNA-Seq identified a minor proportion of 44 genes to exhibit an opposite modulation of expression compared to the microarray results (Figure S2). To substantiate the diverging RNA-Seq results, the differential expression of a subset of 10 genes was verified through RT-qPCR. As shown in Table S5, the expression pattern detected in RT-qPCR confirmed the results obtained from RNA-Seq analysis, though in some cases, the observed level varied. Different outputs from RNA-Seq and microarray approaches have been observed in previous studies that compared these methodologies while analyzing the transcriptome of various organisms [19,20,21,22,23,24]. In all of these studies, RNA-Seq detected more differentially expressed genes than microarray approaches, leading to the identification of additional regulatory pathways. This could be attributed to limitations of microarrays such as cross-hybridization, non-specific-hybridization, and a lower detection range. RNA-Seq overcomes these limitations with its higher sensitivity, specificity, and increased dynamic range of detection.

**Figure 2:**
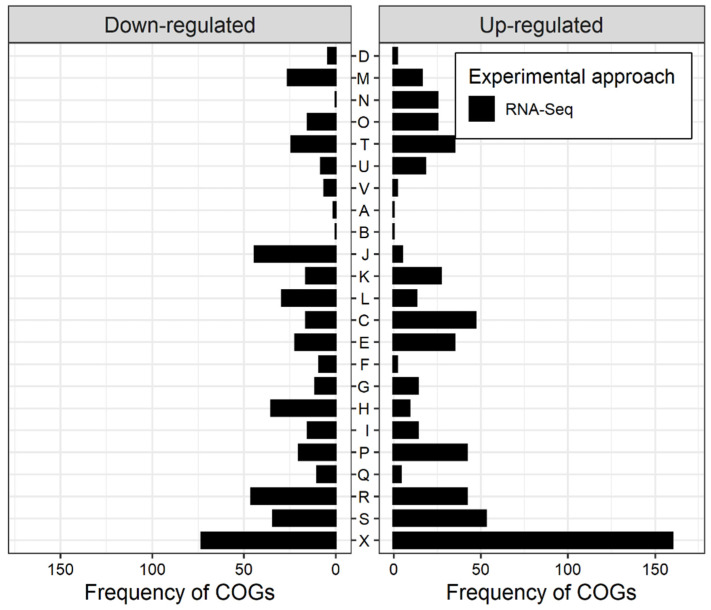
Distribution of cluster of orthologous groups (COGs) of the proteins encoded by DEGs under N_2_-fixing conditions in comparison to growth in ammonium-based growth conditions. Bar plot shows the frequency of each COG category among the genes detected to be up-(**right**) and down- (**left**) regulated by RNA-Seq. The COG categories are D-Cell cycle control, cell division, chromosome partitioning; M-Cell wall/membrane/envelope biogenesis; N-Cell motility; O-Post-translational modification, protein turnover, and chaperones; T-Signal transduction mechanisms; U-Intracellular trafficking, secretion, and vesicular transport; V-Defense mechanisms; A-RNA processing and modification; B-Chromatin structure and dynamics; J-Translation, ribosomal structure, and biogenesis; K-Transcription; L-Replication, recombination, and repair; C-Energy production and conversion; E-Amino acid transport and metabolism; F-Nucleotide transport and metabolism; G-Carbohydrate transport and metabolism; H-Coenzyme transport and metabolism; I-Lipid transport and metabolism; P-Inorganic ion transport and metabolism; Q-Secondary metabolites biosynthesis, transport, and catabolism; R-General function prediction only; S-Function unknown; and X-No COG assigned.

Henceforth in this article, regulation, both up and down, will refer to the differential expression observed under N_2_-fixing conditions. To further comprehend the list of DEGs detected by RNA-Seq, we classified them based on the cluster of orthologous groups (COGs) of the proteins encoded by them (Figure 2). A larger fraction of the genes encoded for proteins that were either poorly characterized or were without any assigned functional category. Among the functionally defined categories, cell motility (N), energy metabolism (C), and inorganic ion transport and metabolism (P) had relatively more enhanced expression than suppression. With this basic classification, it appears that *Azoarcus* is more active in generating energy to fix nitrogen and being motile. To fulfill energy demands, *Azoarcus* probably diverts resources from other metabolic activities. Accordingly, translation machinery (J), coenzyme metabolism (H), and replication and recombination machinery (L) had a higher frequency of down-regulation. When compared to the microarray approach, in general, RNA-Seq detected a higher frequency of enhanced expression and suppression in most of the COG categories (white and black bars in Figure S4, respectively), with a few notable exceptions, such as cell motility (N), energy metabolism (C), and translation machinery (J). Further on in this article, individual pathways will be discussed.

### 3.2. The Higher Sensitivity of RNA-Seq Confirms Known Regulation Patterns

**Figure 3:**
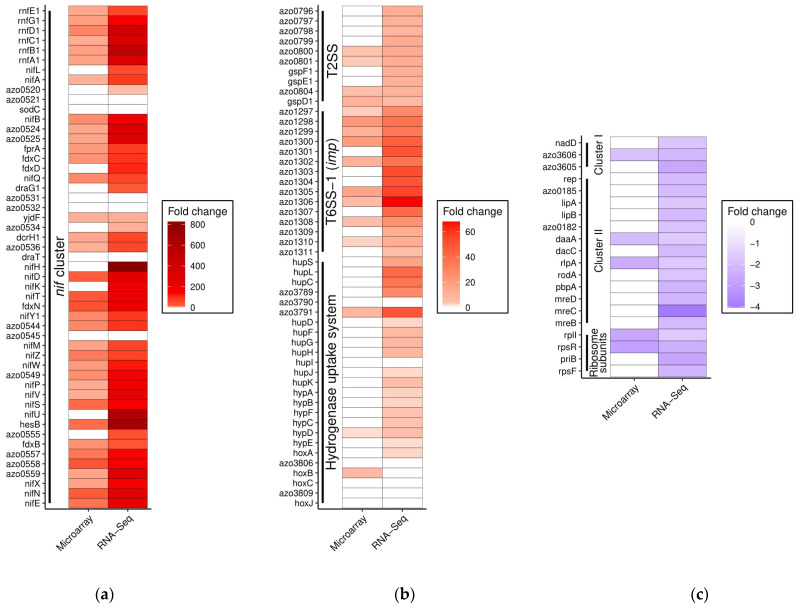
RNA-Seq provides extensive coverage of DEGs in gene clusters under N_2_-fixing conditions. Heat maps indicate the level of up- or down-regulation detected by microarray and RNA-Seq for the genes within clusters previously established to be regulated under nitrogen-fixing conditions: (**a**) nitrogen fixation (*nif*) cluster; (**b**) hydrogenase uptake system, type 6 secretion system-1 (T6SS-1, *imp*), and type 2 secretion system (T2SS); (**c**) ribosome subunits and two clusters of genes with varying functions. Negative fold change values denote down-regulation under N_2_-fixing conditions.

Microarray analysis had already provided a glimpse of gene expression activity during N_2_-fixation. It detected the enhanced transcription of several genes within the nitrogen fixation cluster (*nif*-cluster). This included the genes that are responsible for the regulation of nitrogenase genes, FeMo cofactor synthesis, nitrogenase synthesis, and electron transport. Still, microarrays did not detect the regulation of all of the corresponding genes. Enhanced expression of important genes such as *nifL*, *nifH*, *nifK*, *nifU,* and *fdxD* were not detected. This shortfall was overcome by the robust and more sensitive RNA-Seq method, which detected most of the genes within this cluster to be differentially regulated (Figure 3a). The gene *draG1* was found to be 19-fold up-regulated under N_2_-fixing conditions by using transcriptional reporter gene fusion assays [16]. This was similar to the 24-fold up-regulation detected in the current study. Still, some genes within the cluster were not detected, even by RNA-Seq, to be differentially expressed. One among them, *draT*, was shown to not be responsive to N_2_-fixing conditions by Northern blot analysis [16]. This study proposed that *draT* is part of a polycistronic mRNA along with the downstream genes *azo0536* and *dcrH1*. Contrary to this expectation, we detected both of the downstream genes to be up-regulated in both the RNA-Seq and microarray analyses. A potential secondary promoter might aid in this regulation. RNA-Seq read alignment in this region supported this view (Figure S3). Remarkably, the four genes with the highest predicted fold-changes belonged to the *nif*-cluster. *nifH*, *hesB, nifU,* and *rnfB1* were found to be 823-, 679-, 590-, and 516-fold up-regulated, respectively. This emphasizes the amount of effort taken by the bacterium to fix atmospheric nitrogen.

The *nif*-cluster was not an isolated example, as several other clusters also exhibited a similar pattern validated by RNA-Seq. Among them are the hydrogenase uptake system (*hup* and *hyp* genes) and type 2 as well as one of the two type 6 protein secretion systems (*tss-1 or imp*) (Figure 3b). Type 6 protein secretion systems are particularly interesting, as they can inject effector proteins that may modulate host responses or interbacterial competition, and their gene expression is not known to be induced by N_2_-fixation, except for *A. olearius* BH72 [13]. This was also confirmed by transcriptional reporter gene fusion assays for *azo1299* (=*tssK1*), which demonstrated an 18-fold induction [13], similar to RNA-Seq. Along with the primary detection of regulation, the differential expression rate calculated from this study suggested a relatively stronger up-regulation than microarray analysis for the majority of the overlapping genes (Figure 3a).

We observed a similar trend in the genes that were suppressed, but they were scarce and less pronounced compared to the up-regulated genes (Figure 3c).

### 3.3. The Quest for Alternative Nitrogen Sources during Nitrogen Fixation

**Figure 4:**
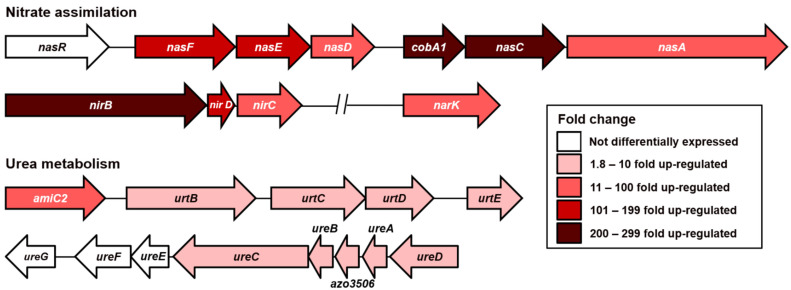
*A. olearius* BH72 prepares itself to assimilate other sources of combined nitrogen under N_2_-fixing conditions. Graphic represents the genomic organization of the genes involved in nitrate assimilation and urea metabolism. Color of each arrow indicates the fold change of up-regulation for the respective gene under N_2_-fixing conditions calculated through the RNA-Seq analysis. Arrows are drawn in proportion to the length, location, and orientation of the corresponding genes within the genome. The two parallel slanted lines indicate a reduction of the large genomic distance between two genes for this representation.

RNA-Seq data revealed the characteristics of strain BH72 during diazotrophic growth that were missed by microarray analyses. Striking among them was the up-regulation of genes encoding proteins to assimilate nitrogen from other sources, even while fixing N_2_. This included assimilatory nitrate reduction, high-affinity ammonium transporters, and urea metabolism. For assimilatory nitrate reduction, *A. olearius* engaged the ABC-transporter NasFED to transport nitrate into the cytoplasm, where it is reduced to nitrite by NasA and NasC. Further, nitrite is commonly reduced to ammonium by the NADH-dependent nitrite reductase complex NirBD. Genes encoding for all of these proteins were up-regulated by *A. olearius*. Additionally, genes encoding channels for nitrate/nitrite transport, *narK,* and *nirC* were also induced. All of these DEGs, except for *narK*, were induced by more than 50-fold; some of them reached over 200-fold (Figure 4). The induction of *nirB* was also verified by RT-qPCR (Table S5). In most bacteria, assimilatory nitrate reduction is controlled by the transcriptional regulators NtrBC and the nitrate-responsive antitermination regulatory protein, NasR. [34,35,36]. This was also true for strain BH72, as a *ntrBC* knockout mutant had a relatively reduced expression of assimilatory nitrate reductase compared to the wild type in the presence of nitrate [37]. Not only the presence of nitrate, but just the absence of ammonium was shown to trigger the expression of assimilatory nitrate reductase [34,36]. A combination of this might have led to the detected enhanced expression in strain BH72. The benefit of expressing this machinery at such high levels is evident from high cost of ATP invested in N_2_-fixation, making potential inputs of more economic nitrogen sources worthwhile.

Less pronounced up-regulation was detected among high-affinity ammonium transporters. *A. olearius* harbors four genes encoding high-affinity ammonium transporters *amtB*, *amtD*, *amtE,* and *amtY*. AmtE is likely a “transceptor” with regulatory functions because the protein combines a typical ammonium transporter at the N-terminus with sensory GGDEF/EAL domains that are commonly involved in the synthesis and hydrolysis of the small signaling molecule, cyclic di-GMP. Consequently, *amtE* expression was enhanced 36-fold. Transcriptional reporter fusion assays have previously shown a similar trend, albeit with a lower fold-change [17]. In an earlier study, *amtB* was shown to be co-transcribed with *glnK* encoding for a P_II_-like protein [15]. Northern blot analysis revealed the enhanced expression of this polycistronic transcript under N_2_-fixing conditions [15], confirming the 9- and 7-fold up-regulation detected by RNA-Seq for both *amtB* and *glnK*, respectively. In addition, the genes involved in urea metabolism were also detected to be up-regulated (Figure 4). Genes encoding for the ABC-transporter complex (AmiC2-UrtBCDE), the urease complex (UreABC), and one accessory protein (UreD) were induced between 2- and 8.8-fold.

Recently, it was proposed [38] that in the absence of inorganic nitrogen, free-living soil diazotrophs might first search for low molecular weight sources such as amino acids. This idea is well substantiated by our transcriptome analysis. RNA-Seq detected the up-regulation of several amino acid transport systems. This included the clusters encoding for the putative polar amino acid transport (*azo2263*-*azo2266*), glutamate/aspartate transport (*glnHPMQ*), and three genes of the branched-chain amino acid transport (*azo1990*, *azo1991*, *azo3730*). The article further proposes that the diazotrophs, while consuming the low molecular weight nitrogen sources, might already start fixing N_2_. N_2_-fixation is conducted as a buffer until it shifts to the breakdown and assimilation of the abundant high molecular weight sources in the soil environment. They propose that the continuous energy requirement of N_2_-fixation might be a drawback in comparison to the assimilation of high molecular weight sources, which needs a one-off high investment. This hypothesis is further supported by previous studies describing pathways that exert tight control on N_2_-fixation and immediately switch-off nitrogenase activity upon detecting a sufficient amount of ammonium [16,39,40].

Even while fixing nitrogen, *Azoarcus* appears to search for low-cost nitrogen sources. A high, constant energy demand and the requirement to maintain a stable microaerobic environment for biological nitrogen fixation might favor this search. Although this model supports the behavior detected in our pure culture experiment, it would be interesting to explore if and how *Azoarcus* modifies this regulation once it is in a symbiotic relationship with plants.

### 3.4. Motility Fostered by N_2_-Deplete Environment–Search for More Favourable Niches?

**Figure 5:**
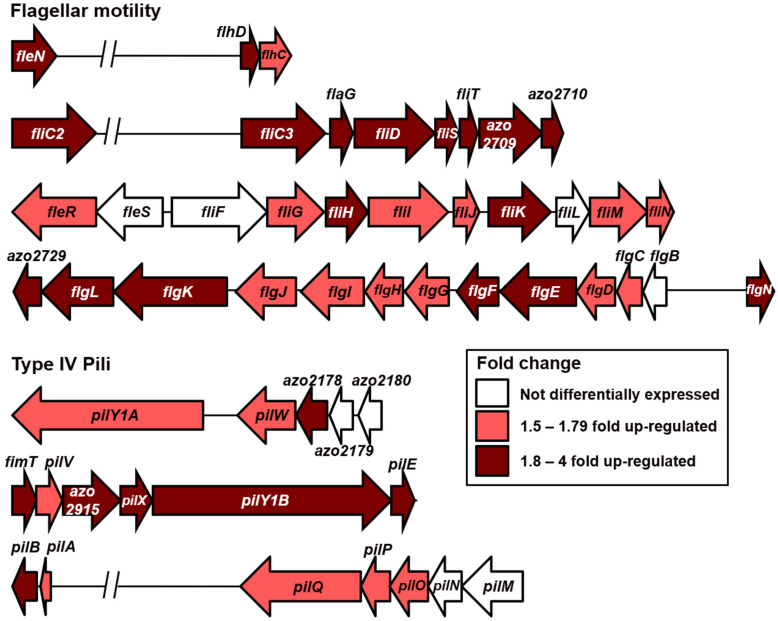
*A. olearius* BH72 shows differential expression of motility-related machineries under N_2_-fixing conditions. Graphic represents the genomic organization of the genes related to the assembly and function of flagellar motility and type IV pili. The color of each arrow indicates the fold change of up-regulation for the respective gene under N_2_-fixing conditions calculated through the RNA-Seq analysis. Arrows are drawn in proportion to the length, location, and orientation of the corresponding genes within the genome. The two parallel slanted lines indicate a reduction of the large genomic distance between two genes for this representation.

As discussed earlier, among the COG categories, cell motility has a higher proportion of enhanced expression than suppression. Strain BH72 contains at least 77 genes annotated to be encoding for proteins related to the assembly and function of flagellar motility and chemotaxis [41]. Out of these, 18 were detected to be significantly up-regulated, with fold-change values between 1.8 and 4 (Figure 5 and Table S6). Looking closer, we identified an additional 17 genes with significant up-regulation (padj < 0.05) but with a lower fold-change, between 1.5 and 1.8 (Table S6 and Figure 5). These genes were located within the flagellar clusters along with several genes that were more than 1.8-fold up-regulated. Hence, they were also considered to be true positives and were included in this analysis. Together, the expression of 35 genes in this module was found to be enhanced during diazotrophic growth. Notable among them were the *flhDC* genes encoding for the transcriptional regulators responsible for the expression of flagellar motility-related genes. Earlier studies have shown that the *flhDC* operon in *E. coli* is regulated by RpoN and that a *rpoN* knockout mutant had a reduced expression of flagellar related genes with a concurrent reduction in motility [42,43]. Next, a gene encoding for the flagellin (*fliC3*) was also found to be induced. Strain BH72 contains three paralogs that encode flagellins. The flagellin encoded by *fliC3* was found to be the major constituent of the flagellar structure and to be responsible for swarming activity [44]. In *E. coli*, *fliC* was found to be up-regulated during N_2_ starvation, and a subsequent CHIP-Seq analysis revealed the binding of NtrC to the promoter sequence of *fliC* [45]. These earlier studies indicated the involvement of the global regulators of nitrogen response, RpoN and NtrC, in the expression of flagellar related genes. These regulators might perform a similar role in strain BH72 under N_2_-fixation.

Similar to the above regulation, the expression of genes encoding the type IV pili machinery was also detected to be induced (Figure 5 and Table S6). Among the fifteen genes that were significantly up-regulated, eight had a fold-change between 1.8 and 4, while the rest had a fold-change between 1.5 and 1.8 (Figure 5 and Table S6). Interestingly, the expression of *pilS,* which is located upstream of the *pilAB* operon, was found to be suppressed by 1.79-fold (Table S6). Previously, in strain BH72, PilS, a NtrC family sensor histidine kinase, was shown to have a strong negative effect on the expression of the pilin PilAB [46]. In the same study, a *pilS* knockout mutant exhibited increased pilin expression under high cell density growth conditions compared to the wild type. Accordingly, its suppression during diazotrophic growth might cause the enhanced expression of the pili structural genes.

Overall, under N_2_-fixation *A. olearius* strain BH72 appears to be more motile compared to N-replete conditions. the organism is likely searching for a suitable growth environment. Here, a suitable condition could depend on at least two variables. The first variable is the concentration of oxygen, as a steady microaerobic environment has to be maintained to create a balance between respiration and the inhibition of nitrogenase. The second factor is the availability of low-cost nitrogen sources, as previously discussed.

### 3.5. Increased Energy Requirements under Diazotrophic Reflected in Transcriptome

Biological nitrogen fixation is an energy-intensive process, as reducing each mole of nitrogen requires at least 16 moles of ATP. Along with fixing N_2_, strain BH72 spends additional energy in search of other nitrogen sources and better environments. The combined energy demand of these activities probably exerts a higher pressure on the energy metabolism of the bacteria. It was observed that an *E. coli* strain, when engineered to fix N_2_, shifted its metabolic flux from the TCA cycle to the pentose phosphate pathway (PPP) and ethanol fermentation. The authors hypothesize that this shift could potentially aid in the rapid response to energy demand [18]. However, *Azoarcus* does not encode glucose transporters [41] and cannot utilize carbohydrates for growth [3]. Hence, in strain BH72, PPP may not be of assistance to rapidly meet the energy demand under N_2_-fixation. Therefore, we looked at other means of carbon assimilation and energy production.

**Figure 6:**
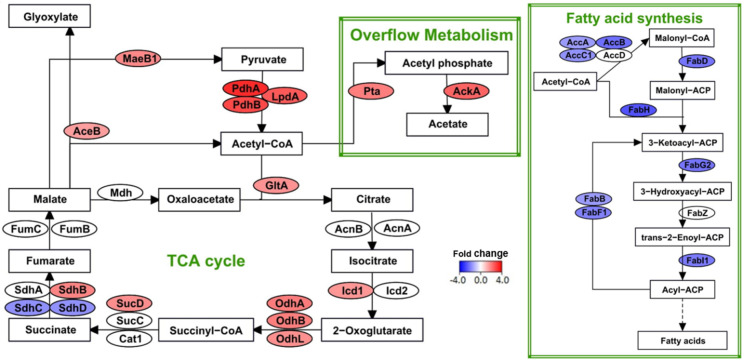
Regulation in carbon metabolism during diazotrophic growth. Flow chart represents the enzymes (ovals) and metabolites (black rectangles) in the tricarboxylic acid (TCA) cycle, overflow metabolism, and fatty acid synthesis pathways. The color of each enzyme indicates the fold change in the expression of the corresponding gene detected by RNA-Seq analysis. Extent of up- or down-regulation during diazotrophic growth are denoted by shades of red and blue, respectively. Green rectangles demarcate the different pathways.

Strain BH72 encodes transporters of dicarboxylic acids [41] and has also been shown to utilize these acids for growth [3]. Thus, we used malate as a carbon source in this study. Within the bacterium, malate is converted to acetyl-Co-A and is then consumed through the TCA cycle. Our previous microarray analysis indicated a general tendency of reduced expression in genes involved in the TCA cycle and in the Embden–Meyerhof–Parnas pathway (EMP). However, RNA-Seq detected an enhanced expression of 12 genes that are involved in malate conversion and the TCA cycle (Figure 6, Table S7). Again, we considered the genes with a fold-change above 1.5 to be significantly regulated (Table S7). The TCA cycle, in addition to energy production, synthesizes 2-oxoglutarate, an important carbon skeleton for nitrogen assimilation. Furthermore, studies on rhizobia have indicated the relationship between several TCA cycle enzymes and N_2_-fixation within root nodules. Specifically, mutants of *S. meliloti* in genes encoding for isocitrate dehydrogenase (*icd*), 2-oxoglutarate dehydrogenase, and citrate synthase (*gltA*) formed root nodules but did not fix nitrogen [47,48,49,50]. Concordantly, RNA-Seq detected the above three genes to be up-regulated in strain BH72.

Surprisingly, two subunits of succinate dehydrogenase (*sdhC* and *sdhD*) were detected to be down-regulated by 1.7-fold. This enzyme is also involved in quinone reduction for oxidative phosphorylation. Additionally, with the exception of a few isolated genes, we did not detect any clear induction or suppression pattern among the genes encoding for the enzymes involved in oxidative phosphorylation. It is most likely that their expression is restricted due to the limited availability of the electron acceptor, oxygen, under microaerobic conditions. In turn, this limits the generation of ATP. Additionally, not regenerating the reducing equivalents could indirectly affect the flux through the TCA cycle. Studies have shown that bacteria with reduced oxidative metabolism or a high redox ratio (NADH/NAD) increases the metabolic flux through the overflow metabolism [51,52,53]. In this pathway, acetyl-Co-A is converted to acetate in a two-step process yielding an ATP molecule by means of substrate-level phosphorylation. Concordantly, in strain BH72, RNA-Seq detected up-regulation for both genes involved in this pathway, *pta* (encoding for phosphate acetyltransferase) and *ackA* (encoding for acetate kinase) (Figure 6). Interestingly, even in the ammonium-grown condition, *pta* was detected to be induced under microaerobic growth when compared to aerobic growth [33].

To reduce the redox ratio, N_2_-fixing bacteria utilize the lipid biogenesis pathways that synthesize poly-ß-hydroxybutyrate (PHB), fatty acids, or both [54,55,56]. Synthesizing these compounds during nitrogen limitations also provides a competitive edge for the bacteria by storing excess carbon for later consumption [55]. It has previously been shown that strain BH72 synthesizes PHB under N_2_-fixing conditions [57]. In the present work, RNA-Seq detected the induction a gene involved in this synthesis, *phbC2*. This gene encodes for PHB synthase, the enzyme that catalyzes the final step in the three-step synthesis of PHB from acetyl-CoA. Moreover, the induction of two probable phasins (*azo0789*, *azo3815*) was also detected. In *B. japonicum*, it was shown that the phasins were more important in PHB accumulation than the metabolic enzymes involved in their synthesis [58]. Concerning fatty acid metabolism, in strain BH72, all but two genes involved in their synthesis were found to be repressed (Figure 6). Nevertheless, these genes were only suppressed between 1.8-and 3-fold. This minor suppression probably helps in maintaining the fatty acid synthesis that is sufficient enough to meet other cellular needs.

The suppression of two enzymes in the pentose phosphate pathway (PPP) was detected. The genes for transketolase (Tkt) and ribose-phosphate diphosphokinase (PrsA) were 1.9-and 4.4-fold suppressed, respectively. As the products of PPP are substrates for several synthetic pathways, its suppression could have ramifications on downstream metabolisms such as those for purine and riboflavin synthesis (discussed later).

Overall, it seems that activities under diazotrophic growth probably demand increased energy production and that strain BH72 responded to the high energy demand by inducing the expression of TCA cycle components. Nevertheless, growth under oxygen-limited conditions restricts the capacity of oxidative phosphorylation, thereby increasing the redox ratio and, in turn, limiting the TCA cycle. Strain BH72 may overcome this hurdle by the induction of overflow metabolism and PHB synthesis.

### 3.6. Suppression of Vitamin Biosynthesis during N_2_-Fixation

**Figure 7:**
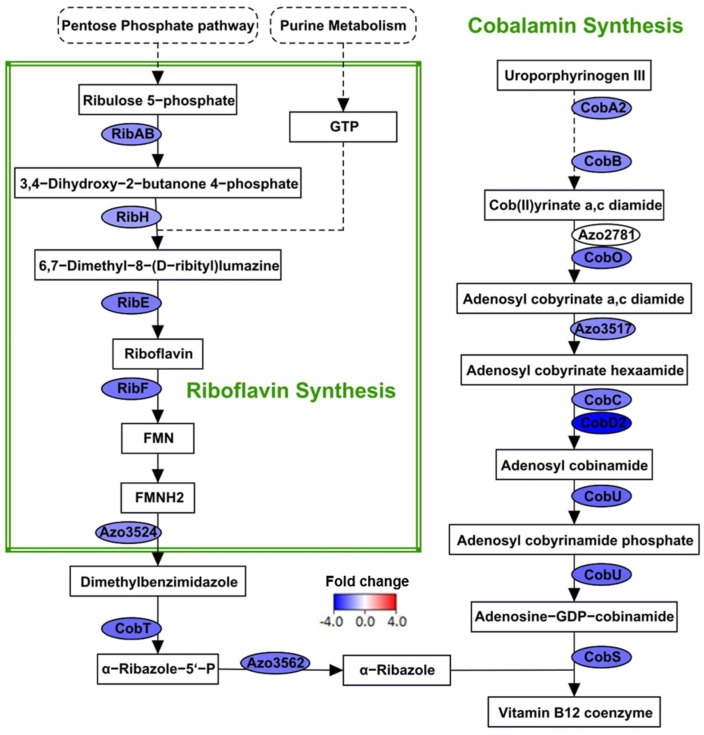
Regulation in riboflavin and cobalamin synthesis under N_2_-fixation. Flow chart represents the enzymes (ovals) and metabolites (black rectangles) in the riboflavin and cobalamin synthesis. The color of each enzyme indicates the fold change in the expression of the corresponding gene detected by RNA-Seq analysis. The extent of down-regulation during diazotrophic growth is denoted by blue shades. The dashed arrow encompasses several enzymatic steps, among which only two corresponding genes were detected to be differentially expressed. Dashed rectangles are pathways that generate the substrates for vitamin biosynthesis. Green rectangles demarcate the different pathways.

Many microorganisms encode the machinery for cobalamin (Vitamin B_12_) synthesis, a cofactor for vital metabolic enzymes. A cobalamin-dependent ribonucleotide reductase is essential for the plant colonization of *S. meliloti* [59]. However, in strain BH72, the genes involved in the synthesis of cobalamin were detected to be suppressed (Figure 7 and Table S8). Knowledge on the relationship between cobalamin synthesis and biological nitrogen fixation is scarce. In *A. vinelandii*, cobalamin synthesis was more dependent on the concentration of cobalt than on the availability of combined nitrogen [60,61]. Additionally, it was able to grow and fix nitrogen even with negligible vitamin B_12_ activity [61].

Similarly, the genes involved in riboflavin (Vitamin B_2_) synthesis were detected to be down-regulated (Figure 7 and Table S8). Riboflavin or its derivatives have been shown to enhance alfalfa root colonization by *S. meliloti* and to also have effects on plant growth promotion traits [62,63]. Considering that we detected the regulation of genes that may be involved in plant colonization under N_2_-fixation, the suppression of riboflavin synthesis appears to be counter-intuitive. Nonetheless, Garcia-Angulo suggests that the paralogs of this module could function independently to synthesize riboflavin for different purposes [64]. Likewise, strain BH72 has another copy of the gene encoding for GTP cyclohydrolase II (*ribA*, *azo3765*), which was induced by 34-fold, which may act complementarily.

Both synthetic pathways are linked; riboflavin is a substrate for cobalamin synthesis. Hence, reduction in riboflavin synthesis might limit the substrate availability for cobalamin synthesis and might be in line with the suppression of the respective synthetic genes. Looking closer, RNA-Seq detected suppression in a sequence of pathways from PPP to cobalamin synthesis. Repression of the genes involved in PPP might reduce the availability of 5-phosphoribosyl 1-pyrophosphate (PRPP), which is a substrate for purine synthesis. Subsequently, we detected genes involved in the purine biosynthesis to be down-regulated (Table S8). This would reduce the production of guanosine triphosphate (GTP). GTP along with ribulose 5-phosphate (from PPP) are substrates for the synthesis of riboflavin. Hence, the limited availability of these two substrates might suppress riboflavin synthesis. As mentioned earlier, lower quantities of riboflavin could impact cobalamin synthesis. Therefore, reduced flux through PPP under N_2_-fixing conditions might be responsible for the repression of riboflavin and cobalamin synthesis. The relationship between GTP availability and riboflavin synthesis has been elucidated in *Bacillus subtilis* [65,66]. In these studies, riboflavin production was enhanced by deregulating the purine pathway and by suppressing ribonucleotide reductase, which competes for the GTP precursor. In summary, the repercussions of N_2_-fixation probably lead to the suppression of vitamin biosynthesis.

### 3.7. Down-Regulation of Ribosomal Genes Corresponding to Decreased Growth Rates

We had previously highlighted DEGs encoding for proteins involved in translation (COG category J). In total, 51 genes encoding for proteins related to ribosome biogenesis and functioning were found to be down-regulated (Table S9). The list also included enzymes that conduct rRNA modification, ribosomal protein modification, and other maturation factors. Considering the energy scenario outlined earlier, bacteria could conserve energy by suppressing these activities, thereby supporting N_2_-fixation, which is in accordance with the reduced growth rate of strain BH72 under N_2_-fixation in comparison to ammonium-grown conditions [15].

### 3.8. Differential Expression of Transcriptional Regulators

RNA-Seq detected 33 DEGs that encode transcriptional factors and response regulators. Out of these, 23 were induced, and 10 were suppressed (Table S10). For eleven of the above-detected response regulators, their cognate histidine kinases were also significantly modulated, albeit with varying strengths (Table S10, directly below the corresponding response regulator). The strongest induction was detected for *nifA* (80-fold) coding for the transcriptional activator of the *nif* genes. *ntrC*, *hoxA,* and *modE* were the other well studied induced regulators. They have an established link to nitrogen metabolism or nitrogen fixation. HoxA, a hydrogenase transcriptional regulatory protein, regulates the expression of structural genes for the hydrogenase uptake system in free-living *B. japonicum* and *Rhodopseudomonas palustris* [67,68,69]. In accordance with this, we also detected the induction of several genes of the hydrogenase uptake system (Figure 3b). The induction of one of those genes, *hupS*, was also substantiated by RT-qPCR (Table S5). Hydrogenase uptake reoxidizes H_2_, the by-product of N_2_-fixation, and regenerates the reducing power. This process increases the efficiency of N_2_-fixation [70]. ModE, the molybdate transport system regulatory protein, represses the expression of the high-affinity molybdate transporter (*modABCD*) genes at high molybdate concentrations [71,72,73]. In strain BH72, the *modE* gene is located in the same cluster as the genes encoding for the transporter (*modA1B1C1*). Correspondingly, RNA-Seq detected the induction of all of these genes. A similar regulation was also detected for *modD*, which was located outside of this cluster. Studies in *Azotobacter vinelandii*, *Paenibacillus* sp. WLY78, and *Rhodobacter capsulatus* have shown that the expression of *modABC* is coordinated with the expression of the Mo-Nitrogenase genes [25,26,74]. Additionally, the loss of *modA* impaired the Mo-Nitrogenase activity of *B. japonicum* within the root nodules of soybean plants under Mo-limiting conditions [75]. Strain BH72 only encodes for the iron–molybdenum cofactor (FeMoCo) dependent nitrogenase. Hence, the regulation of the molybdate transporter system may also be related to nitrogen metabolism. In the future, it would be interesting to discern the roles of other transcriptional regulators, such as *azo1949* and *azo2458*, that are 20-and 5-fold induced, respectively.

Only seven of the above regulators were previously detected by the microarray method (Table S10). Among them, *azo2893* was identified by RNA-Seq to be repressed, while the microarray had indicated an induction. RT-qPCR analysis of the samples used for RNA-Seq substantiated the detected repression (Table S5).

### 3.9. Preparing for Sustained N Starvation

RNA-Seq detected two neighboring genes, *prkA* and *yeaH*, to be induced during diazotrophic growth. PrkA shares 77% of its amino acid sequence identity with the *E. coli* YeaG and contains the same domain organization as a regulatory protein (serine kinase). Additionally, in *E. coli*, both *yeaG* and *yeaH* are present within the same cluster. The promoter of *yeaG* was bound by NtrC under N-starvation [45]. Subsequently, the expression of both of these genes was found to be enhanced under N-starvation [76]. The absence of either of these genes led to the loss of heterogeneity in metabolic activity during prolonged N-starvation [76]. The heterogeneity of the bacterial population during prolonged starvation could help them to overcome the adverse conditions. This mechanism is termed “bet-hedging” [77]. The experimental setup used in this study can probably not be classified as prolonged N-starvation. Nonetheless, strain BH72 appears to be gradually preparing for such situations.

Strong induction was detected for the cluster of three genes (*azo2278*, *azo2279,* and *azo2280*) encoding for hypothetical proteins (490-, 373-, and 443-fold, respectively). Azo2278 has two predicted nitroreductase domains, albeit with very low similarity. Azo2280 seems to be unique to the *Azoarcus* genus, while the first two genes were widespread among prokaryotes. Considering the variety of functions performed by proteins containing nitroreductase domains [78] and the extent of regulation observed in strain BH72, the notion of further investigation into their roles and relationship to N_2_-fixation is compelling.

## 4. Conclusions

**Figure 8:**
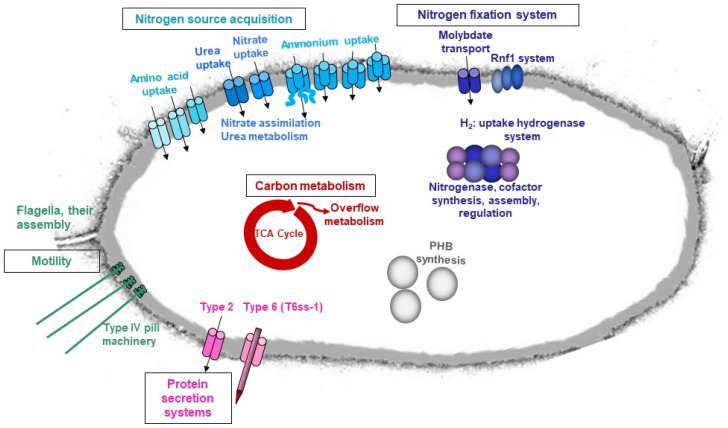
Lifestyle adaptations of *A. olearius* BH72 under limiting N-sources. Image represents pathways that were detected by RNA-Seq to be induced under N_2_-fixing conditions in comparison to ammonium-grown conditions.

With the new data from the reported RNA-Seq analysis, strain BH72 appears to support a N_2_-fixation lifestyle through inducing carbon metabolism and motility and by suppressing translation and vitamin biosynthesis (Figure 8). Still, it is well prepared to utilize alternative economic N-sources such as nitrate, urea, or amino acids (Figure 8). Thus, RNA-Seq has provided a better understanding of the transcriptome during diazotrophic growth and thus of the complex lifestyle of coping with N_2_ as a N-source. Simultaneously, it has ignited the quest to explore the role of non-coding RNA under N_2_-fixation and the regulation of N-assimilation in an endophytic lifestyle. The directional RNA-Seq used in this study revealed the transcription of several antisense and small RNAs. This has been recently reported in several prokaryotes as well [79,80,81]. Identifying their functionalities might aid in obtaining a better picture of the regulatory cascade under N_2_-fixation. This new and vast field necessitates a separate study on its own.

## Data Availability

The reads obtained from the RNA-Seq have been deposited in the NCBI’s Gene Expression Omnibus [28] and are accessible through the GEO Series accession number GSE176473 (https://www.ncbi.nlm.nih.gov/geo/query/acc.cgi?acc=GSE176473; Access date: 1 October 2021).

## Supplementary Materials

The following are available online at https://www.mdpi.com/article/10.3390/microorganisms9091888/s1, Figure S1: Clustering of samples based on RNA-Seq data; Figure S2: Qualitative comparison of genes identified to be differentially expressed by RNA-Seq and microarray analysis in *A. olearius* BH72; Figure S3: Expression of *draT* and downstream genes under N_2_-fixing and ammonium-based growth conditions; Figure S4: Comparison of COG classification of the proteins encoded by DEGs identified by RNA-Seq and microarray under N_2_-fixing conditions in comparison ammonium-based growth conditions; Table S1: List of oligonucleotides used in this study; Table S2: List of barcodes used for multiplexing six samples in RNA-Seq; Table S3: Read alignment statistics obtained for the mapping of RNA-Seq samples; Table S4: List of all differentially expressed genes; Table S5: List of genes verified using RT-qPCR; Table S6: List of differentially expressed genes involved in the assembly and functioning of flagellar structure and Type IV pili; Table S7: List of differentially expressed genes involved in the tricarboxylic acid TCA cycle; Table S8: List of differentially expressed genes involved in cobalamin, riboflavin, and purine synthesis; Table S9: List of genes involved in ribosome biogenesis and functioning that were down-regulated under N_2_-fixing conditions, Table S10: List of differentially expressed transcriptional regulators and histidine kinases.

## Abbreviations

BNF	Biological nitrogen fixation
COGs	Cluster of orthologous groups
DEGs	Differentially expressed genes
EMP	Embden–Meyerhof–Parnas pathway
GTP	Guanosine triphosphate
*nif*-cluster	Nitrogen fixation gene cluster
ORFs	Open reading frames
padj	Benjamini–Hochberg adjusted *p*-value
PHB	poly-ß-hydroxybutyrate
PPP	Pentose Phosphate Pathway
PRPP	5-phosphoribosyl 1-pyrophosphate
RPKM	Reads Per Kilobase per Million mapped reads
SM	Synthetic malate
T2SS	Type 2 protein secretion system
T6SS	Type 6 protein secretion system
TCA	Tricarboxylic acid cycle
