# Supplementary material for: RNA-Seq Provides New Insights into the Gene Expression Changes in Azoarcus olearius BH72 under Nitrogen-Deficient and Replete Conditions beyond the Nitrogen Fixation Process
Source: Microorganisms. 2021 Sep 6;9(9):1888. doi: 10.3390/microorganisms9091888 (PMC8467165; doi:10.3390/microorganisms9091888)
Supplement: Supplementary file 1 [file microorganisms-09-01888-s001.zip › microorganisms-1350862-supplementary.pdf]

microorganisms

RNA-Seq Provides New Insights into the Gene Expression Changes in *Azoarcus olearius* BH72 under Nitrogen-deficient and Replete Conditions beyond the Nitrogen Fixation Process

Shanmugam Solaiyappan Mani and Barbara Reinhold-Hurek

Supplementary Material

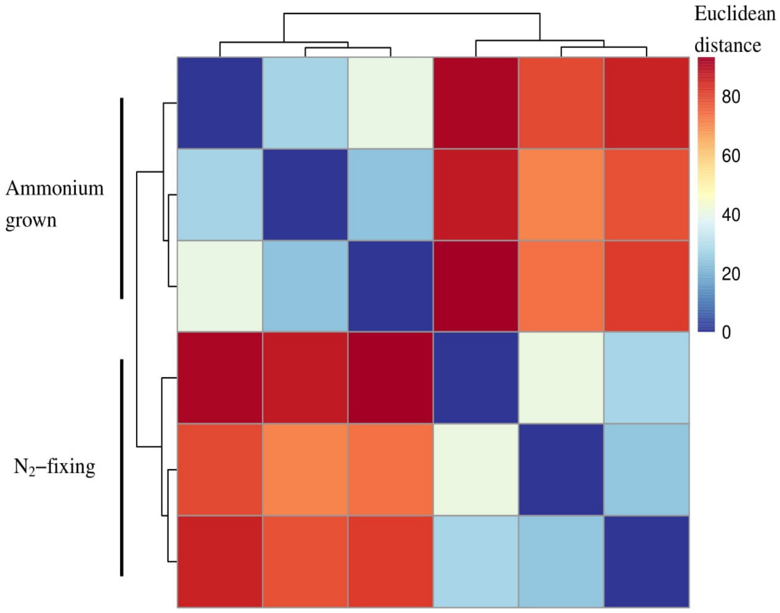

Figure S1. Clustering of samples based on RNA-Seq data. RNA-Seq count data, after variance stabilized transformation, were used to calculate the Euclidean distance. Heat map represents the Euclidean distance between the biological replicates of N_2-fixing and ammonium-based growth conditions. Clustering of the samples is represented by the dendrograms.

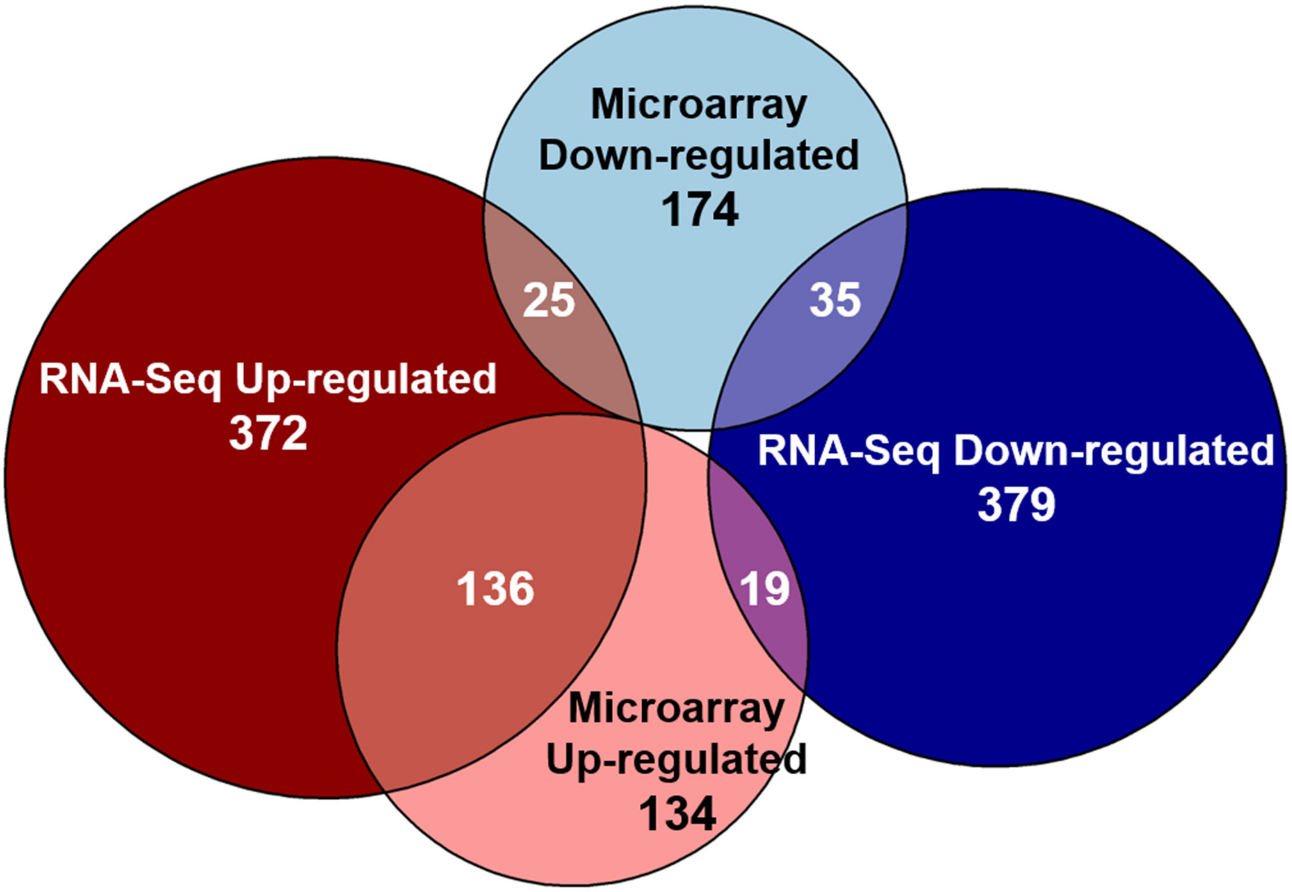

Figure S2. Qualitative comparison of genes identified to be differentially expressed by RNA-Seq and microarray analysis in *A. olearius* BH72. Comparison was done using Euler method, highlighting overlap among the DEGs identified by both the methods. Genes with fold change > 1.8 and adjusted $p < 0.05$ were regarded as DEGs. Up- or down-regulation refer to the enhanced or suppressed expression, respectively, detected under N_2-fixing conditions in comparison to growth in ammonium-based growth conditions. RNA-Seq data from the current study and microarray data from a previous study (Sarkar *et al.*, 2014) were used.
215 genes were detected by both, microarray and RNA-Seq approaches. 171 of these genes showed a similar direction of regulation in both studies and 44 genes exhibited an opposite modulation of expression.

Sarkar, A.; Reinhold-Hurek, B. Transcriptional profiling of nitrogen fixation and the role of NifA in the diazotrophic endophyte *Azoarcus* sp. Strain BH72. *PloS One* **2014**, *9*, e86527.

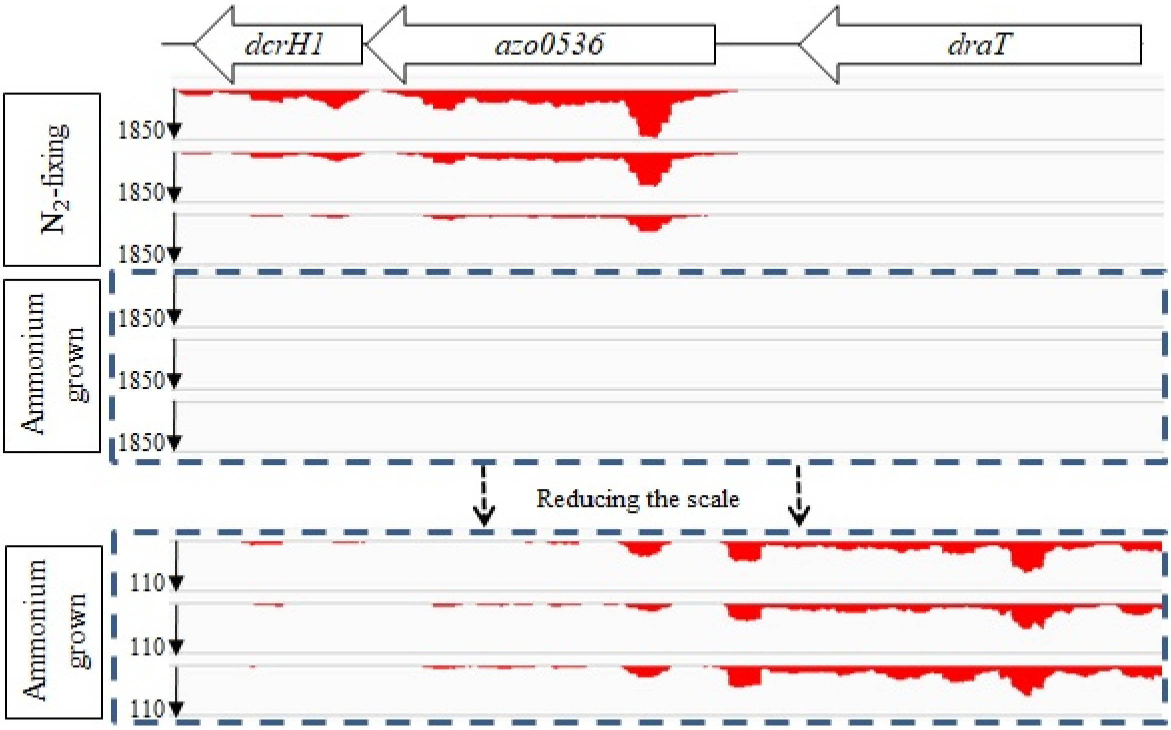

Figure S3. Expression of *draT* and downstream genes under N2-fixing and ammonium-based growth conditions. RNA-Seq reads from the biological triplicates of both experimental conditions were mapped to the genome and a snap shot of a section is shown here. The section includes *draT*, *azo0536* and *dcrH1* encoded in the lagging strand. Each red plot relates to the number of reads mapped to the corresponding base in the genome from each of the six samples. The scale was reduced in the bottom image to visualize the lower expression level detected under ammonium-based growth conditions.

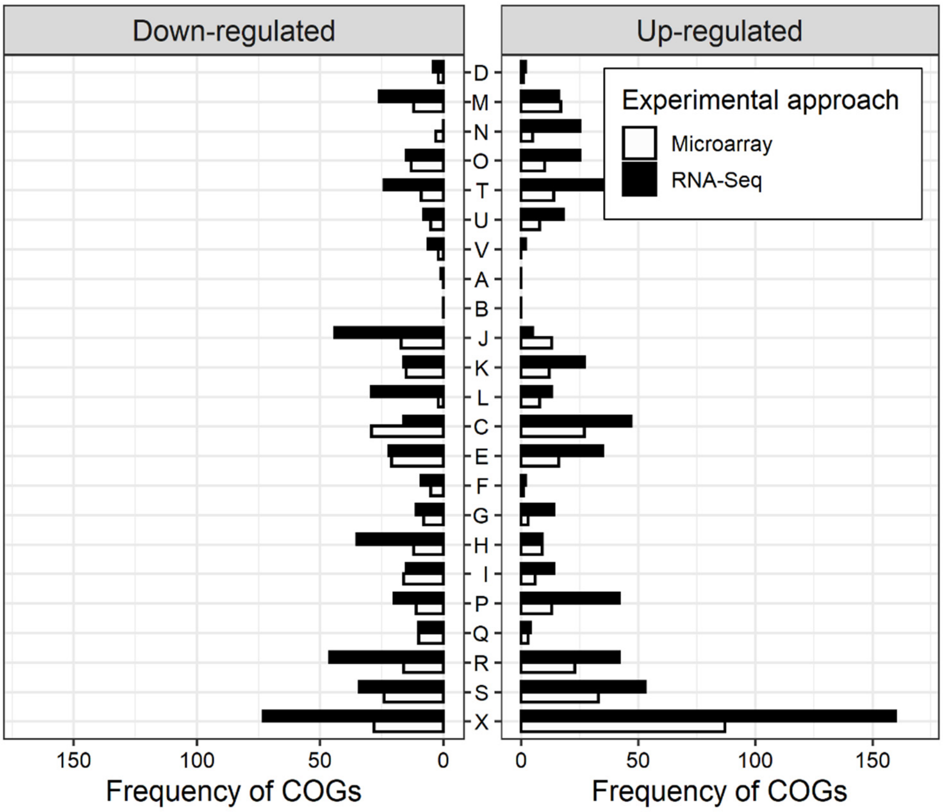

Figure S4. Comparison of COG classification of the proteins encoded by DEGs identified by RNA-Seq and microarray under N_2-fixing conditions in comparison to ammonium-based growth conditions. Bar plot shows the frequency of each COG category among the genes detected to be up-(right) and down- (left) regulated by RNA-Seq (black bars) and microarray (white bars). The COG categories are D-Cell cycle control, cell division, chromosome partitioning; M-Cell wall/membrane/envelope biogenesis; N-Cell motility; O-Post-translational modification, protein turnover, and chaperones; T-Signal transduction mechanisms; U-Intracellular trafficking, secretion, and vesicular transport; V-Defense mechanisms; A-RNA processing and modification; B-Chromatin structure and dynamics; J-Translation, ribosomal structure and biogenesis; K-Transcription; L-Replication, recombination and repair; C-Energy production and conversion; E-Amino acid transport and metabolism; F-Nucleotide transport and metabolism; G-Carbohydrate transport and metabolism; H-Coenzyme transport and metabolism; I-Lipid transport and metabolism; P-Inorganic ion transport and metabolism; Q-Secondary metabolites biosynthesis, transport, and catabolism; R-General function prediction only; S-Function unknown; X-No COG assigned.

The categories cell motility (N) and energy metabolism (C) were found to be less frequently suppressed in the current study. Genes encoding for proteins related to translation machinery (J) were less frequently up-regulated and were found to be more repressed than in the previous study.

.

Table S1. List of oligonucleotides used in this study

Oligo name	Sequence	Annealing temperature (°C)
5′ TruSeq adapter	AATGATACGGCGACCACCGAGATCTACAC-NNNNNNNN[1]ACACTCTTTCCCTACACGACGCTCTTCCGATCT	-
3′ TruSeq adapter	CAAGCAGAAGACGGCATACGA-GATNNNNNNNN[2]GTGACTGGAGTTCAGACGTGTGCTCTTCCGATCT	-
0305RTFor<br>0305RTrev	TGGTCTGGATCACAAGCACT<br>TCCAGGCGACGGGTATAGA	64.7
1175RTFor<br>1175RTrev	CACCGTGCTGTGCGAAGAA<br>CGACAGGTCTTCTGCGGTCT	64.7
2264RTFor<br>2264RTrev	ATCGCATCTTCGGCGTCTAT<br>GCCATTCGGTCATCTTCACG	
2687RTFor<br>2687RTrev	GGTCTGCTTGCCGGTTTGAT<br>CTGCGAAACAGCCATACGGT	
2837RTFor<br>2837RTRev	ACCAAGTACGACACCACCCA<br>ATCCTTGGTGGAGAAGAACGG	64.7
2893RTFor<br>2893RTrev	CGGACCCTCGACCAGTATTT<br>CCACGGTCTGGTTACCCTT	
3398RTFor<br>3398RTrev	ATACCATCAAACCCGGTGCG<br>TTCTGACCCTTGTGACCACG	64.7
3409RTFor<br>3409RTrev	GCGTGGACGAATTGAACGGA<br>CGCACCTTGCCAATCTGACT	64.7
3512RTFor<br>3512RTrev	GATCAAGTTCACCCTCGACTGG<br>GATCTCCACGTCCAGCCCTT	
3786RTFor<br>3786RTrev	CAAAGGCAACTACATCCTCGC<br>CACAGGAACCCCAGGAGATG	64.7
3956RTFor<br>3956RTrev	TACTCGAAATCCAGGGTTGCC<br>GCCACATTGCAGCGTGAAG	64.7

[1] NNNNNNNN – position of i5 barcode
[2] NNNNNNNN – position of i7 barcode

Table S2. List of barcodes used for multiplexing six samples in RNA-Seq.

No.	Condition	Replicate	i5 barcode	i7 barcode
1	Nitrogen-fixing	I	ACGATGTG	CGGCTATG
2	Nitrogen-fixing	II	GACAAATG	CGGCTATG
3	Nitrogen-fixing	III	TGGACGGG	TCCGCGAA
4	Ammonium-based growth	I	ATTGACTG	CGGCTATG
5	Ammonium-based growth	II	AACCATGG	TCCGCGAA
6	Ammonium-based growth	III	GATATCGG	TCCGCGAA

Table S3. Read alignment statistics obtained for the mapping of RNA-Seq samples.

Condition	N_2-fixing			Ammonium-based growth		
**Biological replicate**	**I**	**II**	**III**	**I**	**II**	**III**
No. of input reads	10602624	10822876	10717584	10586006	11803381	11561465
Total no. of aligned reads	8809284	9204604	9019811	9252710	10462742	10138782
Percentage of aligned reads (compared to no. of input reads)	83.09	85.05	84.16	87.41	88.64	87.69
Total no. of uniquely aligned reads	8447237	8862835	8683542	8680911	9855265	9629912
Percentage of uniquely aligned reads (in relation to all aligned reads)	95,89	96,29	96,27	93,82	94,19	94,98

Table S4. List of all differentially expressed genes with fold change more than 1.8. Provided as excel file.

Table S5. List of genes verified using RT-qPCR.

Gene	Name	Product	RT-qPCR		RNA-Seq		Microarray	
			Fold Change[1]	*p*-value	Fold Change[1]	padj[2]	Fold Change[1]	*p*-value
azo0305	*paaB*	phenylacetic acid degradation protein PaaB	1.7	0.12			- 5.4	0.001
azo1175	*nirB*	assimilatory nitrite reductase (NAD(P)H) large subunit precursor (EC 1.7.1.4)	101	5.6E-04	249	1.2E-46	- 2.1	0.026
azo2264		amino acid ABC transporter membrane protein 1, PAAT family (TC 3.A.1.3.-)	23.7	6.3E-04	15.3	5.5E-17	- 3.2	0.003
azo2687		hypothetical secreted protein	-2.5	0.046	-5.4	8E-07		
azo2893		DNA-binding protein Fis	-2.3	8.5E-03	-2.8	5.6E-07	+ 2.5	0.013
azo3398	*rplO*	LSU ribosomal protein L15P	1.5	0.28			- 4.2	0.003
azo3409	*rpmC*	LSU ribosomal protein L29P	1.0	0.973			+ 6.1	0.018
azo3512	*ssuA*	putative periplasmic-binding protein	16.3	3.15E-03	20.8	1.2E-17		
azo3786	*hupS*	ferredoxin hydrogenase, small chain	20.6	7.80E-05	16.6	2.4E-37		
azo3956		conserved hypothetical protein	-3.4	0.040	-5.6	7.6E-06		

[1] fold change - fold change of up- (+) or down- (-) regulation of genes under N_2-fixation as compared to ammonium-based growth conditions

[2] padj - Benjamini–Hochberg adjusted *p*-value

Table S6. List of differentially expressed genes involved in the assembly and functioning of flagellar structure and Type IV pili.

	Gene	Name	Product	RNA-Seq		Microarray	
				Fold Change[1]	padj	Fold change[1]	*p*-value
**Flagellar motility**	*azo1106*	*fleN*	hypothetical flagellar related protein FleN	1.82	0.001		
	azo1446	*flhD*	flagellar transcriptional activator FlhD	2.05	0.0106		
	azo1447	*flhC*	flagellar transcriptional activator FlhC	1.77	0.0092		
	azo1450	*cheY2*	putative chemotaxis response regulator	1.54	0.0136		
	azo1452	*cheW2*	CheW protein	1.57	0.0047		
	azo1453	*tsr*	putative serine chemoreceptor protein	1.63	0.0002	- 2.5	0.032
	azo1459	*cheV2*	probable chemotaxis protein CheV-like	1.64	3E-05		
	azo1461	*cheZ*	chemotaxis protein CheZ	1.54	2E-05		
	azo2693	*fliC2*	flagellin	2.17	1E-06		
	azo2704	*fliC3*	flagellin	2.57	9E-08		
	azo2705	*flaG*	probable flagellar protein	2.00	0.0002		
	azo2706	*fliD*	flagellar hook-associated protein	2.29	2E-08		
	azo2707	*fliS*	flagellar protein FliS	2.16	3E-06		
	azo2708	*fliT*	hypothetical flagellar related protein FliT	2.12	2E-06		
	azo2709		hypothetical protein	2.18	5E-08		
	azo2710		conserved hypothetical flagellar related protein	2.51	4E-09		
	azo2712		diguanylate cyclase with PAS/PAC sensor	-2.38	8E-18		
	azo2714	*fleR*	sigma-54 dependent response regulator	1.73	4E-06		
	azo2717	*fliG*	flagellar motor switch protein FliG	1.70	1E-08		
	azo2718	*fliH*	flagellar assembly protein FliH	1.80	1E-04		
	azo2719	*fliI*	ATP synthase	1.68	0.0005		
	azo2721	*fliK*	putative flagellar hook-length control protein FliK	2.32	8E-09		

	azo2723	*fliM*	flagellar motor switch protein FliM	1.63	0.0017		
	azo2724	*fliN*	flagellar motor switch FliN	1.62	0.0011		
	azo2729		hypothetical secreted protein	1.89	0.0003		
	azo2730	*flgL*	flagellar hook-filament junction protein 3	2.33	2E-08		
	azo2731	*flgK*	flagellar hook-filament junction protein 1	2.40	1E-05		
	azo2732	*flgJ*	peptidoglycan hydrolase	1.63	0.0039		
	azo2733	*flgI*	flagellar P-ring protein precursor	1.63	0.0015		
	azo2734	*flgH*	flagellar L-ring protein precursor	1.56	0.0078		
	azo2735	*flgG*	flagellar basal-body rod protein FlgG	1.59	0.0115		
	azo2736	*flgF*	flagellar basal-body rod protein FlgF	1.95	4E-09		
	azo2737	*flgE*	FlgE protein	1.95	2E-08		
	azo2738	*flgD*	probable basal-body rod modification protein FlgD	1.66	0.0014		
	azo2739	*flgC*	flagellar basal-body rod protein FlgC	1.66	0.0007		
	azo2743	*flgN*	putative chaperon of flagellar synthesis	2.07	1E-11		
**Type IV pili**	*azo1790*	*rpoN2*	RNA polymerase, sigma 54 subunit, RpoN/SigL	3.10	6E-05	+ 3.8	0.003
	azo2175	*pilY1A*	putative type 4 pilus biogenesis protein	1.64	2E-06	+ 2.4	0.002
	azo2177	*pilW*	putative type 4 pilus biogenesis protein	1.64	1E-05		
	azo2178		probable type 4 fimbrial biogenesis related transmembrane protein	2.06	3E-13		
	azo2913	*fimT*	putative pre-pilin like protein	1.95	0.0085		
	azo2914	*pilV*	putative prepilin-like protein	1.76	0.0473		
	azo2915		conserved hypothetical secreted protein	2.52	0.0005		
	azo2916	*pilX*	putative Tfp pilus assembly protein	2.71	3E-06		
	azo2917	*pilY1B*	putative Tfp pilus assembly protein	3.43	1E-11	+ 3.4	0.007
	azo2918	*pilE*	putative prepilin like protein	3.75	1E-13		
	azo3354	*pilB*	Type IV pilus assembly protein	1.81	4E-12	- 2.4	0.021
	azo3355	*pilA*	tfp structural protein	1.74	2E-07		
	azo3357	*pilS*	two component system sensor protein	-1.79	1E-06		

azo3646	*pilQ*	probable type 4 fimbrial biogenesis protein	1.73	0.0001
azo3647	*pilP*	putative type 4 fimbrial biogenesis protein	1.58	1E-07
azo3648	*pilO*	probable type 4 fimbrial biogenesis protein	1.68	4E-05

[1] fold change - fold change of up- (+) or down- (-) regulation of genes under N_2-fixation as compared to ammonium-based growth conditions

Table S7. List of differentially expressed genes involved in the tricarboxylic acid (TCA) cycle.

Gene	Name	Product	RNA-Seq result	
			Fold Change[1]	padj
azo0821	*maeB1*	probable malate dehydrogenase (oxaloace-tate-decarboxylating) (NADP+)	1.84	0.002371
azo1147	*icd1*	isocitrate dehydrogenase (NADP) (EC 1.1.1.42)	1.61	0.030575
azo1159	*aceB*	AceB protein	1.50	0.004426
azo1371	*lpdA*	dihydrolipoamide dehydrogenase (EC 1.8.1.4)	2.60	0.002448
azo1372	*pdhB*	probable dihydrolipoamide acetyltransferase	2.83	0.000349
azo1373	*pdhA*	pyruvate dehydrogenase	3.32	4.67E-05
azo1549	*sdhC*	succinate dehydrogenase subunit C (EC 1.3.5.1)	-1.71	0.000484
azo1550	*sdhD*	succinate dehydrogenase subunit D (EC 1.3.5.1)	-1.71	6.95E-06
azo1552	*sdhB*	succinate dehydrogenase subunit B (EC 1.3.5.1)	1.89	1.05E-13
azo1554	*gltA*	citrate synthase (EC 2.3.3.1)	1.69	0.014766
azo1555	*odhA*	2-oxoglutarate dehydrogenase E1 component (EC 1.2.4.2)	2.05	1.04E-16
azo1556	*odhB*	2-oxoglutarate dehydrogenase E2 component (EC 2.3.1.61)	2.01	2.35E-21
azo1557	*odhL*	dihydrolipoamide dehydrogenase (EC 1.8.1.4)	1.71	9.97E-12
azo3332	*sucD*	succinyl-CoA synthetase (ADP-forming) alpha subunit (EC 6.2.1.5)	1.77	4.07E-06

[1] fold change - fold change of up- (+) or down- (-) regulation of genes under N_2-fixation as compared to ammonium-based growth conditions

Table S8. List of differentially expressed genes involved in cobalamin, riboflavin and purine synthesis.

	Gene	Name	Product	RNA-Seq	
				Fold Change[1]	padj
Cobalamin synthesis	*azo3517*		adenosylcobyric acid synthase (glutamine-hydrolysing) (EC 6.3.5.10)	-1.85	2E-06
	azo3518	*cobD2*	adenosylcobinamide-phosphate synthase (EC 6.3.1.10)	-3.89	5E-30
	azo3519	*cobC*	cobalamin biosynthesis protein	-2.07	7E-06
	azo3550	*cobB*	cobyrinate a,c-diamide synthase (EC 6.3.5.-) / hydrogenobyrinic acid a,c-diamide synthase (glutamine-hydrolysing) (EC 6.3.5.9)	-1.84	2E-08
	azo3551	*cobA2*	Uroporphyrin-III C-methyltransferase	-1.83	2E-07
	azo3552	*cobO*	cob(I)yrinic acid a,c-diamide adenosyltransferase (EC 2.5.1.17)	-2.18	1E-06
	azo3557	*cobU*	adenosylcobinamide kinase (EC 2.7.1.156)	-2.41	0.0028
	azo3560	*cobT*	nicotinate-nucleotide-dimethylbenzimidazole phosphoribosyltransferase	-2.33	2E-17
	azo3561	*cobS*	cobalamin-5'-phosphate synthase (EC 2.7.8.26)	-2.28	4E-06
	azo3562		putative alpha-ribazole phosphatase	-2.17	9E-06
Riboflavin synthesis	*azo0318*	*ribH*	6,7-dimethyl-8-ribityllumazine synthase (EC 2.5.1.9)	-1.51	0.0032
	azo0319	*ribAB*	GTP cyclohydrolase II	-1.75	0.001
	azo0320	*ribE*	riboflavin synthase	-2.07	2E-07
	azo1206	*ribF*	riboflavin kinase (EC 2.7.1.26) / FMN adenylyltransferase (EC 2.7.7.2)	-2.13	1E-06
	azo3524		cob(II)yrinic acid a,c-diamide reductase (EC 1.16.8.1)	-1.80	1E-05
Purine synthesis	*azo0923*	*ndk*	nucleoside diphosphate kinase (EC 2.7.4.6)	-1.66	0.0004
	azo1087	*surE*	3'-nucleotidase (EC 3.1.3.6) / exopolyphosphatase (EC 3.6.1.11) / 5'-nucleotidase (EC 3.1.3.5)	-1.95	5E-10
	azo1269	*purT*	phosphoribosylglycinamide formyltransferase 2 (EC 2.1.2.-)	-2.40	7E-13
	azo1378	*purE*	phosphoribosylaminoimidazole carboxylase	-2.06	2E-05
	azo1379	*purK*	phosphoribosylaminoimidazole carboxylase (EC 4.1.1.21)	-2.14	3E-07
	azo1473	*adk*	Adenylate kinase (EC 2.7.4.3)	-1.75	0.0007
	azo2842	pykA	pyruvate kinase (EC 2.7.1.40)	-1.63	7E-05
	azo2873	*purC*	phosphoribosylaminoimidazolesuccinocarboxamide synthase	-1.57	0.0002
	azo2894	*purH*	IMP cyclohydrolase (EC 3.5.4.10) / phosphoribosylaminoimidazolecarboxamide formyltransferase (EC 2.1.2.3)	-2.07	1E-09

azo3124		phosphoribosylglycinamide formyltransferase (EC 2.1.2.2)	-1.49	0.0033
azo3136	*purM*	phosphoribosylformylglycinamidine cyclo-ligase (EC 6.3.3.1)	-1.60	0.01
azo3742	*purU*	formyltetrahydrofolate deformylase (EC 3.5.1.10)	-2.22	1E-13
azo3953	*gmk*	guanylate kinase (EC 2.7.4.8)	-1.66	4E-06

[1] fold change - fold change of up- (+) or down- (-) regulation of genes under N_2-fixation as compared to ammonium-based growth conditions

Table S9. List of genes involved in ribosome biogenesis and functioning that were down-regulated under N_2-fixing conditions.

Gene	Name	Product	RNA-Seq		Microarray	
			Fold Change[1]	padj	Fold change[1]	*p*-value
azo0086	*efp*	translation elongation factor P (EF-P)	-3.001	9.5E-20	+ 1.8	0.047
azo0100	*fmt*	methionyl-tRNA formyltransferase (EC 2.1.2.9)	-3.002	1.16E-13		
azo0109	*yihZ*	D-tyrosyl-tRNA(Tyr) deacylase	-2.416	1.56E-05		
azo0122		probable Fe-S cluster redox enzyme	-2.517	4.19E-15		
azo0316	*rhlE1*	putative ATP-dependent RNA helicase	-2.258	0.000612		
azo0380	*argS*	arginyl-tRNA synthetase (EC 6.1.1.19)	-2.067	2.82E-05		
azo0718	*rpsF*	SSU ribosomal protein S6P	-2.233	2.35E-06		
azo0720	*rpsR*	SSU ribosomal protein S18P	-2.375	0.000514	- 2.8	0.021
azo0785		glycyl-tRNA synthetase alpha chain (EC 6.1.1.14)	-2.088	1.4E-11		
azo0872		conserved hypothetical protein	-1.805	0.000139		
azo0924		23S rRNA m(2)A-2503 methyltransferase (EC 2.1.1.-)	-1.998	5.98E-11		
azo1009		conserved hypothetical protein	-2.161	2.3E-09		
azo1045		pseudouridine synthase A	-1.942	0.000234		
azo1083	*pheS*	phenylalanyl-tRNA synthetase, alpha subunit (EC 6.1.1.20)	-1.943	0.00039	- 2.4	0.02
azo1100		23S rRNA m(5)U-1939 methyltransferase (EC 2.1.1.-)	-2.632	9.94E-12		
azo1101		ribosomal large subunit pseudouridine synthase E (EC 5.4.99.-)	-2.354	1.54E-08		
azo1135	*rpmG*	LSU ribosomal protein L33P	-2.865	3.8E-10		
azo1136	*rpmB*	LSU ribosomal protein L28P	-2.401	0.000113		
azo1312		tRNA (uracil-5-)-methyltransferase (EC 2.1.1.35)	-2.948	6.31E-20	- 2.1	0.01
azo1327	*hflX2*	GTP-binding protein HflX (EC 3.1.5.-)	-1.993	0.000205		
azo1385		conserved hypothetical protein	-3.930	6.96E-21		
azo1465		tRNA pseudouridine synthase C (EC 5.4.99.-)	-2.074	0.000319		
azo1518	*orn*	oligoribonuclease	-2.016	0.000353		
azo1521	*rpmE*	LSU ribosomal protein L31P	-2.872	1.79E-05		
azo1522		conserved hypothetical protein	-2.484	3.68E-08		
azo1621	*rpmF*	LSU ribosomal protein L32P	-3.412	2.44E-08		

azo2105	*truB*	tRNA pseudouridine synthase B (EC 4.2.1.70)	-2.138	0.032934		
azo2109		conserved hypothetical protein	-2.430	4.97E-07	+ 2.0	0.013
azo2190	*rpsT*	SSU ribosomal protein S20P	-2.252	0.009043	+ 2.3	0.017
azo2602	*infA1*	bacterial translation initiation factor 1 (bIF-1)	-2.440	0.001521		
azo2759	*rpsI*	SSU ribosomal protein S9P	-2.774	1.08E-12		
azo2760	*rplM*	LSU ribosomal protein L13P	-2.429	1.11E-07		
azo2815		tRNA/rRNA methylase	-1.929	0.000276	+ 2.2	0.016
azo2884	*ksgA*	dimethyladenosine transferase (EC 2.1.1.-)	-1.816	0.00062		
azo2889		Nucleotidyltransferase	-2.157	4.25E-06		
azo2892	*dusB*	conserved hypothetical tRNA-dihydrouridine synthase	-2.577	9.88E-08		
azo2898	*rpsP*	SSU ribosomal protein S16P	-2.291	0.000296		
azo2899	*rimM*	16S rRNA processing protein RimM	-2.139	1.39E-06		
azo3131	*miaA*	MiaA protein	-2.578	5.31E-09		
azo3140	*pcnB*	probable poly(A) polymerase	-2.600	7.38E-10		
azo3167	*rplU*	LSU ribosomal protein L21P	-2.338	0.001031	+ 1.8	0.021
azo3168	*rpmA*	LSU ribosomal protein L27P	-2.142	4.09E-05		
azo3225	*rpsU*	SSU ribosomal protein S21P	-2.783	1.44E-08		
azo3405	*rplE*	LSU ribosomal protein L5P	-1.882	0.030542	- 3.1	0.006
azo3406	*rplX*	LSU ribosomal protein L24P	-2.116	0.013893	- 4.3	0.019
azo3422	*rpsL*	SSU ribosomal protein S12P	-1.922	0.001653		
azo3522	*rhlE3*	putative ATP-dependent RNA helicase	-3.747	1.3E-12	- 1.8	0.022
azo3610	*def2*	probable peptide deformylase	-2.759	1.87E-09		
azo3695	*trpS*	tryptophanyl-tRNA synthetase (EC 6.1.1.2)	-2.109	1.93E-07		
azo3991		ribonuclease P protein component (EC 3.1.26.5)	-2.372	1.85E-06		
azo3992	*rpmH*	LSU ribosomal protein L34P	-2.906	3.93E-09		

[1] fold change - fold change of up- (+) or down- (-) regulation of genes under N_2-fixation as compared to ammonium-based growth conditions

Table S10. List of differentially expressed transcriptional regulators and histidine kinases.

Gene	Name	Product	RNA-Seq		Microarray	
			Fold Change[1]	padj	Fold change[1]	*p*-value
azo0704	*creB*	two component transcriptional regulator, winged helix family	-2	0.0003		
azo0703[H]	*creC*	catabolite repression sensor kinase for PhoB	-2.44	6.00E-05		
azo0735	*ntrC*	nitrogen regulation protein NR(I)	5.6	1.00E-08		
azo0736[H]	*ntrB*	nitrogen regulation protein NR(II)	2.09	0.0037		
azo1490		conserved hypothetical signaling protein	2.39	0.0004		
azo1491	*divK*	response regulator receiver protein	2.15	0.003		
azo1584		putative two-component hybrid sensor and regulator	2.26	1.00E-10	6.3	0.009
azo2455	*rcp2*	response regulator receiver protein	2.11	0.001		
azo2456[H]	*cph1*	putative two-component system sensor protein	1.62	0.0079		
azo2749	*vsrD2*	probable transcriptional regulator, LuxR family	1.82	6.00E-05		
azo2748[H]		putative sensory box histidine kinase	1.51	0.0004		
azo2770	*yfhA*	two component, sigma54 specific, transcriptional regulator, Fis family	-2.44	0.0015		
azo2768[H]	*yfhK*	putative two-component system sensor kinase	-4.15	2.00E-08		
azo3093	*kdpE*	two component system transcriptional regulatory protein	2.22	0.0053		
azo3094[H]	*kdpD2*	probable sensor for high-affinity potassium transport system	1.61	0.0254		
azo3191	*atoC*	probable acetoacetate metabolism regulatory protein	1.88	7.00E-09		
azo3192[H]		putative two component sensor histidine kinase	1.48	0.0443		
azo3499		response regulator receiver modulated diguanylate cyclase/phosphodiesterase with PAS/PAC sensor(s)	1.81	7.00E-10	1.8	0.04
azo3498[H]		histidine kinase (EC 2.7.13.3)	1.58	0.0034		
azo3615	*ompR3*	two component transcriptional regulator, winged helix family	-1.85	9.00E-06		
azo3616[H]	*envZ3*	putative sensor histidine kinase	-2.02	6.00E-05		
azo3665		response regulator receiver modulated diguanylate cyclase/phosphodiesterase	2.2	9.00E-05	2.2	0.017
azo3666[H]		putative sensor histidine kinase	2.93	0.0004	2.6	0.014

azo3685		putative hybrid sensor and regulator protein	-2.29	9.00E-05		
azo3805	*hoxA*	hydrogenase transcriptional regulatory protein	2.57	3.00E-06		
azo3945		putative hybrid sensor and regulator protein	-2.03	0.0002		
azo3946		putative two component transcriptional regulator	-2.04	1.00E-06		
azo3947[H]		putative two component sensor protein	-2.12	1.00E-05		
azo0212	*prp*	transcriptional regulator, AsnC family	5.76	6.00E-05		
azo0243	*uidR*	transcriptional regulator, TetR family	-2.09	4.00E-05	-2.1	0.001
azo0519	*nifA*	nif-specific regulatory protein	80.33	4.00E-70	1.8	0.042
azo0644		putative regulatory protein	-3.21	0.0016		
azo0679	*nodD*	transcriptional regulator, LysR family	11.42	1.00E-10		
azo1015		transcriptional regulator, LysR family	-2.01	1.00E-09		
azo1118		transcriptional regulator, AraC family	2.95	0.0011		
azo1320		transcriptional regulator, AraC family	2	0.045		
azo1936		transcriptional regulator, AraC family	1.84	9.00E-05		
azo1949		transcriptional regulator, AraC family	20.51	3.00E-07		
azo2261	*eutR*	transcriptional regulator, AraC family	4.59	0.0005		
azo2262	*pdhR*	transcriptional regulator, GntR family	11.58	5.00E-05		
azo2304		transcriptional regulator, MerR-family	2.02	0.0001		
azo2458		putative transcriptional regulator, LuxR family	5.1	5.00E-09		
azo2893		DNA-binding protein Fis	-2.77	6.00E-07	2.5	0.013
azo3840	*modE*	putative molybdenum transport protein	3.57	1.00E-05	3.5	0.008

[1] fold change - fold change of up- (+) or down- (-) regulation of genes under N_2-fixation as compared to ammonium-based growth conditions

[H] Cognate histidine kinases of two-component regulators with padj < 0.05

1. Sarkar, A.; Reinhold-Hurek, B. Transcriptional profiling of nitrogen fixation and the role of NifA in the diazotrophic endophyte *Azoarcus* sp. strain BH72. *PLoS One* **2014**, *9*, e86527.
